# Tamoxifen and Endometrial Cancer: A Janus-Headed Drug

**DOI:** 10.3390/cancers12092535

**Published:** 2020-09-07

**Authors:** Günter Emons, Alexander Mustea, Clemens Tempfer

**Affiliations:** 1 Department of Obstetrics and Gynecology, Georg-August-University, 37075 Göttingen, Germany; 2 Department of Gynecology and Gynecological Oncology, University Hospital, 53127 Bonn, Germany; mustea@ukbonn.de; 3 Department of Obstetrics and Gynecology, Ruhr University, 44625 Herne, Germany; clemens.tempfer@ruhr-uni-bochum.de

**Keywords:** tamoxifen, endometrial cancer, selective estrogen receptor modulator

## Abstract

**Simple Summary:**

Tamoxifen, an antiestrogen, is a potent drug to treat and prevent hormone dependent breast cancer. As it has low toxicity and is widely available, tamoxifen has become one of the most frequently prescribed anticancer drugs worldwide. A major side effect of tamoxifen is to increase the risk of uterine corpus cancer (endometrial cancer). This happens after long-term (>2 years) application, especially in postmenopausal women with preexisting pathologies in the uterus. On the other hand, tamoxifen is an efficacious treatment for certain forms of advanced endometrial cancer, thus making it a Janus-headed drug that can support the development of endometrial cancer on one hand and be used as a remedy for this disease on the other. This article reviews the clinical data on these controversial effects of tamoxifen and the possible explanations.

**Abstract:**

Tamoxifen is a selective estrogen receptor modulator used for the treatment and prevention of estrogen receptor (ER)—positive breast cancer. However, tamoxifen increases the risk of endometrial cancer (EC) by about 2–7 fold, and more aggressive types of EC with poor prognoses are observed in tamoxifen users. On the other hand, tamoxifen is an efficacious treatment for advanced or recurrent EC with low toxicity. The differential agonistic or antagonistic effects of tamoxifen on ERα are explained by the tissue-specific expression profiles of co-activators and co-repressors of the receptor. The estrogen-agonistic effect of tamoxifen in endometrial cancers can also be explained by the expression of G-protein coupled estrogen receptor 1 (GPER-1), a membrane-bound estrogen receptor for which tamoxifen and other “antiestrogens” are pure agonists.

## 1. Introduction

Tamoxifen is a selective estrogen receptor modulator (SERM) originally developed as a contraceptive or morning-after pill [1]. It failed in this indication before its antiestrogenic properties were discovered to be efficacious in the treatment of estrogen receptor (ER)—positive breast cancer [1,2]. In the last few decades, tamoxifen has become one of the most frequently prescribed anticancer drugs worldwide, also used for the chemoprevention of breast cancer for women at high risk [3]. 

In 1985, Killackey et al. suggested a possible link between tamoxifen use and the development of endometrial cancer (EC) [4], a claim substantiated by a series of later studies [3,5], with an increased risk of EC ranging from 1.5 to 6.9-fold in tamoxifen users [6]. In addition, the ECs in tamoxifen users often belong to less favorable subtypes and have relatively poor prognoses [6]. While tamoxifen has an antiestrogenic effect in the breast, it can act as a weak estrogen agonist on the endometrium. Tamoxifen-associated changes include endometrial hyperplasia, atypia, and malignancy [1,2,3,4,5]. 

On the other hand, tamoxifen has been successfully used as treatment in advanced or recurrent EC [7]. It is suggested to be the preferred second-line hormonal therapy after first-line progestin treatment, particularly in patients with endometrial tumors and a positive estrogen receptor status, achieving response rates of 10–53% [7]. Thus, tamoxifen is a Janus-headed drug regarding EC: on the one hand, it increases the risk for the development of this disease, and it represents a remedy for certain forms of this cancer on the other.

This short narrative review will summarize our knowledge on the incidence and prognosis of EC in women using tamoxifen for the treatment or chemoprevention of breast cancer, to characterize the potential risk factors for the development of EC and analyze measures for the prevention and early detection of tamoxifen-associated EC. 

We also review the data available regarding the application of tamoxifen as a therapy for recurrent or advanced EC. Finally, we discuss the possible molecular mechanisms of tamoxifen-associated EC and its mode of action in the treatment of EC. 

## 2. Methods

We performed a systematic literature search of PubMed from 2010 to May 2020 using the terms “tamoxifen” and “endometrial cancer”, identifying 450 articles. The most actual systematic reviews and meta-analyses (*n* = 12) regarding different aspects of the topic and the most relevant original studies were used as cited in “references”. 

When the systematic reviews referred to pertinent papers published before 2010, these were acquired and cited, respectively. As there are excellent existing systematic reviews (*n* = 12) on each of the different aspects of tamoxifen and EC, the aim of the present paper was not another systematic review but to provide an up-to-date synopsis of these different facets.

## 3. Incidence of Endometrial Cancer in Tamoxifen Users

### 3.1. Adjuvant Therapy of Invasive Breast Cancer with Tamoxifen

NSABP-B14 was a large, double-blind, placebo-controlled trial to determine the efficacy of adjuvant tamoxifen in patients with early ER-positive breast cancer. A 7.5-fold increase in EC in the tamoxifen arm vs. placebo was observed. The annual incidence rates of EC were 1.6/1000 (tamoxifen) and 0.2/1000 (placebo) [8]. In the Oxford overview of 2005, analyzing data of breast cancer patients who had taken tamoxifen for 5 years (*n* = 7512) and adjusted controls (*n* = 7502), an annual incidence rate for uterine cancer of 0.19%/year was found for the tamoxifen group as compared to 0.06%/year in the control patients [9]. In a meta-analysis of 21,457 patients with early breast cancer from 20 trials comparing about 5 years of adjuvant tamoxifen use with no tamoxifen, the Early Breast Cancer Trialists’ Collaborative Group found a rate ratio of 2.40 (SE, 0.32) for EC for tamoxifen users of all ages [10]. For women younger than 45 years taking tamoxifen, the rate ratio for EC was 1.04 (SE, 0.62; *p* = 1.00); for those 4554 years, it was 1.75 (SE, 0.55; *p* = 0.25); and only for those 55–69 years did tamoxifen significantly increase EC risk (rate ratio = 2.96 ± 0.44; *p* = 0.00002) [10]. There were nine deaths in the tamoxifen group versus one in the control group from uterine (excluding cervix) cancer, during a mean follow up of 10 years [10]. All-cause mortality was substantially reduced by tamoxifen treatment (rate ratio, 0.78; SE, 0.03; *p* = 0.0001) due to the decrease in breast cancer deaths from breast cancer recurrence [10]. 

Between 1982 and 1992, the Swedish Breast Cancer Group randomized 4610 postmenopausal women with early-stage invasive breast cancer to two or 5 years of adjuvant tamoxifen treatment. The risk of EC tripled during treatment (HR, 3.33; 95% CI, 1.33–8.34) but subsequently decreased after its cessation (HR, 1.50; 95% CI, 0.91–2.45) [11]. 

The Adjuvant Tamoxifen: Longer Against Shorter (ATLAS) trial randomized 12,894 women with early breast cancer to receive adjuvant tamoxifen treatment for 5 or 10 years. In the 10 years group, the cumulative EC risk during Years 5–14 was 3.1% (EC-related mortality, 0.4%) versus 1.6% (mortality, 0.2%) in women receiving tamoxifen for only 5 years [12]. Only 10% of the patients in the ATLAS trial were premenopausal [12]. 

In the Adjuvant Tamoxifen Treatment: Offer More? (aTTom) trial 6953 women with early breast cancer were randomized to 5 or 10 years of adjuvant tamoxifen treatment. There were 102 cases of EC with 10 years versus 45 cases with 5 years of tamoxifen treatment (RR = 2.20; 95% CI, 1.31–2.34) with 37 (1.1%) versus 20 (0.6%) EC-specific deaths [13]. 

A meta-analysis of the risk of endometrial malignancy in extended tamoxifen therapy reported a RR of 2.29 (95% CI, 1.5–3.2) with reference to the standard five years of therapy. The cumulative risk of endometrial malignancy increased from 1.5 to 3.2 percent with extended therapy [5]. 

Aromatase inhibitors (AI) have partly or completely replaced tamoxifen as the adjuvant endocrine therapy of choice in postmenopausal women. A meta-analysis on 31,920 postmenopausal women with ER-positive early breast cancer randomized to 5 years of tamoxifen, 5 years of AI, or 2–3 years of tamoxifen and 2–3 years of AI found fewer cases of EC with AI than tamoxifen (10 year incidence, 0.4% vs. 1.2%; RR, 0.33; 95% CI, 0.21–0.51), with five versus nine EC-related deaths [14]. 

Chlebowski et al. compared EC rates in hormone receptor-positive breast cancer patients in a SEER-affiliated tumor registry; 5303 women were treated with AI, 5155 with tamoxifen, 3787 with AI and tamoxifen (switchers), and 2819 with no adjuvant endocrine therapy [15]. EC incidence was lowest in the AI-only group (0.95/1000 person years), followed by the no-endocrine-therapy group (0.98/1000 person years). The highest EC rate was in the tamoxifen-only group (1.83/1000 person years). The HR for AI versus tamoxifen was 0.52 (95% CI, 0.31–0.87; *p* = 0.01) [15].

The Tamoxifen and Exemestane Trial (TEXT) and Suppression of Ovarian Function Trial (SOFT) randomized trials compared adjuvant AI (exemestane) plus ovarian suppression with adjuvant tamoxifen plus ovarian suppression for a period of 5 years in premenopausal women with hormone receptor-positive breast cancers. There were two and five cases of EC in the exemestane (*n* = 2359) and tamoxifen (*n* = 2358) groups, respectively [16]. 

### 3.2. Chemoprevention of Invasive Breast Cancer with Tamoxifen

The Cochrane meta-analysis of risk-reducing medications for primary breast cancer included six studies with 50,927 healthy women with an elevated risk for breast cancer. Women receiving prophylactic tamoxifen had a higher risk of developing EC (RR, 2.26; 95% CI, 1.52–3.38) [17]. 

The systematic review on “Medication use for the risk reduction of primary breast cancer in women” by the U.S. Preventive Services Task Force found a RR of 2.25 (1.17–4.41) for EC in women taking tamoxifen versus placebo or 4 (1–8) more/1000 women, vs. a placebo rate of 0.62/1000 [18]. The risks of developing EC were higher in older than in younger women and returned to normal after the end of tamoxifen medication [19]. 

## 4. Prognosis of Tamoxifen-Associated EC

Curtis et al. compared data from 39,451 breast cancer patients initially treated with tamoxifen with those from the general breast cancer population from the Surveillance, Epidemiology, and End Results (SEER) program (primary diagnosis from 1980 to 2000). They detected an increased observed versus expected ratio (O/E) of 2.17 (95% CI = 1.95–2.41) for uterine cancer in women that had received tamoxifen [20]. For uterine malignant mixed Mullerian tumors (MMMTs), the relative risk was significantly higher than that for adenocarcinomas (O/E = 4.62, 95% CI = 3.20–6.46 versus 2.07, 95% CI = 1.85–2.32). The excess absolute risk for MMMTs was 1.4 and for adenocarcinoma 8.4 per 10,000 women/year [20]. The cumulative mortality (15 years after initial treatment) from uterine corpus cancer in breast cancer patients that had taken tamoxifen was very low (0.37%; 95% CI = 0.23–0.51) [20]. 

The Dutch Study Group on Tamoxifen Associated Malignancies (TAMARISK) analyzed the data of 332 patients with corpus cancer after breast cancer. In long-term tamoxifen users (*n* = 161), a higher proportion of non-endometrioid ECs (serous ECs and MMMTs) than that in non-users was observed (32.7% vs. 14.4%; *p* = 0.004). Furthermore, the proportion of International Federation of Gynecology and Obstetrics (FIGO) stage III and IV tumors was 20% (tamoxifen) vs. 11.3% (no tamoxifen) (*p* = 0.049) [21] Three year uterine corpus cancer-specific survival was worse for long-term tamoxifen users than for non-users (82% vs. 93%; *p* = 0.0001) [21]. The genomic profile of tamoxifen-associated EC, as compared with that of tumors that developed in breast cancer patients without tamoxifen treatment, was found to depend on morphologic subtype and not on previous tamoxifen exposure [22]. 

Bland et al. [23] found an association of tamoxifen use for at least 60 months with high-risk uterine histological subtypes (Grade 3 endometrioid, serous, or clear cell EC) compared to no tamoxifen use. Ngo et al. [24] observed more carcinosarcomas in women treated with tamoxifen and a worse prognosis for EC patients previously treated for breast cancer, particularly if they received tamoxifen. Pierce et al. [25] found no difference in progression-free (PFS) or overall survival (OS) between women with serous EC with or without preceding breast cancer or tamoxifen exposure. 

## 5. Risk Factors for Tamoxifen-Associated EC

The risk of tamoxifen-associated EC was not correlated with the daily dose of the drug but with the duration of use and cumulative dosage [5,11,12,13]. The risk of EC was higher in postmenopausal and older patients than in premenopausal and younger women and increased with body weight [5,6,26]. Breast cancer patients (regardless of hormone receptor status) have a significantly increased risk of secondary EC, even if they do not receive tamoxifen [27]. Older age and higher body mass seem to be shared risk factors for EC in the general population as well as in breast cancer survivors. Independently of tamoxifen use, postmenopausal breast cancer patients have a 20% prevalence of endometrial proliferative disorders—including hyperplasia, polyps, atypical hyperplasia (2%), and even EC (0.6%)—according to the assessment of the endometrium prior to adjuvant tamoxifen treatment [6,28]. 

The highest risk for tamoxifen-associated EC is therefore found in postmenopausal women with higher body mass and endometrial pathology before tamoxifen treatment [5,6], while the risk for premenopausal women is very low [5,10].

## 6. Prevention of Tamoxifen-Associated EC

Systemic and local progestogens have a protective effect against the development of endometrial hyperplasia, having been used for the conservative therapy of atypical endometrial hyperplasia and well-differentiated endometrioid EC [3,29]. The South West Oncology Group (SWOG) performed a randomized trial of the efficacy of intermittent medroxyprogesterone acetate (MPA) in tamoxifen users that had normal endometrium at baseline. A total of 149 patients received tamoxifen alone, and 147, tamoxifen plus 10 mg of MPA for 14 days every 3 months. After 2 years, 89 women on tamoxifen alone and 80 on the combined treatment were evaluable for endometrial pathology. There were four women with proliferative endometrium and one with simple hyperplasia in the tamoxifen arm versus one with proliferative endometrium in the combined arm. After 5 years, only one new proliferative event was detected (one patient with proliferative endometrium in the tamoxifen + MPA arm). The authors concluded that the observed event rates in both arms were much lower than planned and that a negative pre-tamoxifen evaluation of the endometrium was associated with an extremely low risk of developing tamoxifen-associated EC or precursors [3]. Breast cancer progression or death were observed in 15% (tamoxifen only) versus 10% (tamoxifen plus MPA) of patients [3]. 

In a systematic Cochrane review, four randomized controlled trials (543 patients) of the intrauterine use of levonorgestrel for endometrial protection in women receiving adjuvant tamoxifen for breast cancer were analyzed. Patients on tamoxifen had either a levonorgestrel intrauterine system (LNG-IUS), releasing 20 µg of LNG/day plus endometrial surveillance, or endometrial surveillance alone (controls). The LNG-IUS reduced the incidence of benign endometrial polyps and hyperplasia. However, only six cases of endometrial hyperplasia were observed. The LNG-IUS increased abnormal vaginal bleeding at 12 and 24 months, but by 60 months, no cases of abnormal bleeding were observed in either group. None of the trials were sufficiently powered to detect significant differences in EC incidence, breast cancer recurrence, or death between the LNG-IUS and control groups [30]. 

In a placebo-controlled randomized trial, Davis et al. ascertained the effect of 850 mg of metformin twice daily on the endometrium of postmenopausal women (*n* = 102) with hormone receptor-positive breast cancer taking tamoxifen. The endometrial thickness after one year was significantly lower in the metformin group. These women had also significantly greater weight and waist circumference reductions and improvement of metabolic parameters. However, in neither the metformin nor the placebo group were endometrial atypia or EC observed [31]. 

Lazzeroni et al. evaluated the reduction of the tamoxifen dose from 20 to 5 mg/day in patients with ductal carcinoma in situ (DCIS) or other preinvasive disease of the breast. After 5 years of follow-up, the women on low-dose tamoxifen had a marked decrease in the incidence of DCIS or invasive breast cancers as compared to the placebo group, but no increase in EC incidence [32]. 

## 7. Endometrial Surveillance in Tamoxifen Therapy

Endometrial thickness assessed by transvaginal ultrasound (TVUS) has been used for many years for the early detection of atypical endometrial hyperplasia and EC during tamoxifen treatment [5]. A recent systematic review of four trials involving 926 patients found that to detect one case of EC in women on adjuvant tamoxifen, 332 TVUS examinations and 56 endometrial biopsies had to be performed [5]. All the patients with endometrial malignancy had vaginal bleeding and were postmenopausal [5]. Tamoxifen increases endometrial thickness due to subendometrial gland hypertrophy, and this can occur in the absence of any atypical features [5,33]. Studies on the routine surveillance of tamoxifen users (5 years) have not shown any benefit but, rather, an increase in harmful side effects (complications of surgery, unnecessary anxiety, overtreatment of asymptomatic endometrial changes on TVUS, and reduction of adherence to tamoxifen) [5,33]. This is reflected by most guidelines on the diagnosis and treatment of EC [5,34], which do not recommend routine TVUS for asymptomatic women on tamoxifen. These patients should be counseled about the risk of tamoxifen-associated EC and the lack of benefit and the risks of routine surveillance. They should be advised to report any abnormal gynecological symptoms (vaginal bleeding or discharge) immediately, to allow for a prompt assessment by TVUS, biopsy, or hysteroscopy and curettage [5,33,34]. 

In a recent retrospective analysis from the Republic of Korea of 821 breast cancer patients on tamoxifen in whom endometrial biopsies had been performed, seven cases (0.9%) of atypical endometrial hyperplasia and seven patients (0.9%) with EC were found. Of the patients, 77.2% had normal endometrium and 21% had endometrial polyps. The mean age at biopsy was 48.0 ± 7.6 years and body mass index 23.0 ± 3.2 kg/m^2^. The authors found that women with breast cancer on tamoxifen may have a higher risk of endometrial pathology when they had lower parity, increased endometrial thickness on TVUS, or abnormal vaginal bleeding [35]. The authors do not indicate why an endometrial biopsy was performed, and univariate and multivariate analyses were not performed [35]. Therefore, it does not seem justified to change the current practice of endometrial surveillance and counseling on the basis of this study [33,35]. 

Another retrospective study from the Republic of Korea analyzed endometrial biopsies from 284 premenopausal patients treated with tamoxifen [36]. Hysteroscopic evaluation and biopsy were performed if the endometrial thickness was greater than 10 mm, new or enlarged polyps were detected at yearly TVUS, or a patient had vaginal bleeding with or without an increase in endometrial thickness. The mean age of the patients was 45.0 ± 4.7 years and the body mass index 22.5 ± 3.8 kg/m^2^. A total of 284 biopsies were performed. Atypical endometrial hyperplasia was found in seven patients (2.5%), and EC, in five patients (1.8%). Endometrial polyps were found in 114 women (40.1%). All the patients with EC and five of the seven patients (71%) with atypical endometrial hyperplasia had abnormal uterine bleeding, which turned out to be the only significant factor upon uni- and multivariate analysis [36]. 

Garuti et al. [28] investigated 146 postmenopausal breast cancer patients who were candidates to receive tamoxifen. They found 31 cases (21%) of baseline pathology, of which 2.7% were atypical (three atypical hyperplasias, one EC). The uteri with atypical endometria were extirpated, and polyps with or without simple hyperplasia were fully removed by hysteroscopic procedures before the start of tamoxifen treatment. In seven out of 27 patients with baseline endometrial abnormalities (26%), recurrent endometrial pathology was found during 5 years of tamoxifen administration. No case of atypical hyperplasia or EC was observed. Of 114 patients with normal endometrium before the start of tamoxifen therapy, 43 (31.5%) developed endometrial pathologies (polyps and non-atypical hyperplasia) during treatment. No atypical lesion was observed. This study highlights the importance of the detection of endometrial pathologies before the initiation of tamoxifen treatment. 

## 8. Tamoxifen as Treatment for EC

Endogenous and exogenous estrogens are most important risk factors for type 1 ECs, as are estrogen medication, nulliparity, early menarche, late menopause, and obesity [7,34]. Tamoxifen has been used for the treatment of advanced or recurrent EC since the 1980s, at that time considered to act primarily as an antiestrogen by blocking the estrogen receptor α (ERα) [37]. In 2001, a phase II trial by the Gynecologic Oncology Group (GOG) involving 68 eligible patients with advanced or recurrent EC was performed with 40 mg of tamoxifen/day. Three complete (4%) and four (6%) partial responders were observed [37]. However, 50% of the patients had Grade 3 tumors and at least 20% had Type 2 tumors that were not candidates for endocrine therapy due to the lack of ER [38,39,40]. The authors concluded that tamoxifen demonstrated modest activity at best and did not warrant further investigation as a single agent in this population [37]. 

In a subsequent phase II trial, the GOG combined tamoxifen with intermittent medroxyprogesterone acetate (MPA) or megestrol acetate. The rationale was that tamoxifen induced the expression of progesterone receptors, downregulated by continuous progestin treatment, via its estrogen-agonistic activity. Overall response rates of 33% (tamoxifen plus MPA) [41] and 27% (tamoxifen plus megestrol acetate) [42] were observed. The median overall survival was 1314 months [41,42]. Estrogen receptor α measured in metastatic EC tissue prior to hormonal therapy was statistically significantly related to clinical response to daily tamoxifen and intermittent MPA [38]. 

In 2010, a systematic Cochrane review did not find evidence that hormonal therapy improved survival in patients with recurrent or primarily advanced EC [43]. This review, however, looked at randomized controlled trials focusing on overall survival or 5 years disease-free survival benefits and did not include phase II trials or observational studies. Furthermore, due to insufficient and heterogeneous data, the authors were not able to pool data and perform meta-analyses. In addition, the role of hormone receptor expression was not explored [43]. A later systematic review tried to overcome the limitations of the Cochrane analysis and found an objective response rate for tamoxifen in first-line treatment of 21.4% ± 12.1% and clinical benefit rate of 57.1 ± 10.4%. In second-line treatment, an objective response rate of 20.6 ± 13% and a clinical benefit rate of 36.3 ± 16% were calculated [40]. 

For the combination of SERMs (mostly tamoxifen) plus progestins, an objective response rate of 24.2 ± 8.3% and clinical benefit rate of 32.3 ± 15.8% were observed [40]. 

For all hormonal treatments, the response rates were higher in ER+ (26.5%) and PGR+ (35.5%) disease and lower in ER- (9.2%) or PGR- (12.1%) tumors. The response rates in second-line treatment were significantly higher when a response had been achieved in first-line HT [40]. 

Another systematic review on antiestrogen treatment in endometrial cancer found response rates for tamoxifen monotherapy ranging from 10% to 53% and for combined tamoxifen/progestin treatment of 19–58% [7]. The authors concluded that tamoxifen alone or in combination with a progestogen should be the preferred second-line hormonal treatment. The response rates were comparable to those for first-line progestogen treatment, and toxicity was low. The efficacy of tamoxifen therapy could be further improved by selecting patients with endometrioid EC and positive ER and/or PR status, preferably based on an actual biopsy [7]. 

## 9. Molecular Mechanisms of Tamoxifen in EC

Tamoxifen was originally considered to be an antiestrogen before its estrogen-agonistic activities on various organs, including the uterus and the breast, became evident [1,2]. This led to the paradigm “tamoxifen is an estrogen antagonist in the breast and an agonist in the endometrium” [1,2]. This concept was elegantly supported by experiments from the group of Jordan. They examined the effects of tamoxifen on an estrogen-dependent human breast cancer cell line and an estrogen-dependent human EC cell line xenotransplanted into athymic mice. In the same animal, tamoxifen treatment stimulated the endometrial cancers while inhibiting the breast cancer transplant [44]. This suggested that not the host metabolism but rather tissue-specific actions of tamoxifen led to the contrasting effects of the drug [44]. It was later shown that the recruitment of co-activators and co-repressors of the ER might determine cell type-specific cellular responses to tamoxifen [6,45]. 

Tamoxifen is metabolized to a variety of molecules, including intermediates that could form protein or DNA adducts and cause DNA damage [6]. Tamoxifen is a strong liver carcinogen in rats. The analysis of tamoxifen-DNA adducts in endometrial tissues from women with breast cancer taking tamoxifen has not provided convincing results. The risk of women treated with tamoxifen developing hepatocellular cancer is minimal, and the formation of tamoxifen-DNA adducts in endometrial tissues occurred at extremely low levels and in only a few patients [6]. 

The Dutch TAMARISK group investigated whether ECs that developed after long-term (>2 years) tamoxifen treatment for breast cancer was genetically different from ECs occurring without exposure to tamoxifen. An analysis of endometrioid ECs, serous ECs, and carcinosarcomas found neither more nor different genomic aberrations between tumors that developed after prolonged tamoxifen use and those that developed in the absence of tamoxifen [22]. 

Genes typically mutated in EC include *p53* (type 2 EC), *PTEN* (phosphatase and tensin homology), and the DNA mismatch repair family genes. In the majority of cases, patients exposed to tamoxifen had similar mutation rates as non-exposed females with EC [6]. 

The long-term treatment of estrogen-dependent breast cancer eventually results in tamoxifen resistance, even after an initial remission of the tumors [1,2]. It could be shown that tamoxifen not only lost its estrogen-antagonistic activity but could even stimulate the growth of some of these tumors [1,2]. Apart from the well-characterized ERα and ERβ, a third estrogen receptor has been identified in a variety of tissues, which is located in the plasma membrane and mediates certain rapid actions of estrogens. [6,46]. This seven-transmembrane receptor (GPR-30) is coupled to G-protein and activates the epidermal growth factor/MAPK pathway [6,46]. As it is potently activated by estrogens, it has been renamed to G-protein coupled estrogen receptor 1 (GPER1) [46]. 

Apart from estrogens, tamoxifen and fulvestrant—considered to be a pure antiestrogen—and a number of phytoestrogens and endocrine disruptors act as agonists [46]. The expression of GPER1 is increased in breast cancers with acquired tamoxifen resistance [46]. 

Vivacqua et al. showed that tamoxifen antagonized the activation of ERα by estradiol in Ishikawa EC cells and, at the same time, stimulated the mitogenic signaling pathways through GPER1, leading to cell proliferation [47]. When the expression of GPER1 was suppressed, tamoxifen inhibited estrogen-induced proliferation by blocking ERα [47]. GPER1 was found to be overexpressed in endometrial cancers where ER and PR were downregulated, and in high-risk endometrial cancers with lower survival rates [48].

Ignatov et al. [49] demonstrated a significant stimulation of endometrial cancer cell lines by tamoxifen in vitro through GPER1. In vivo, they found a significant correlation between GPER1 expression and tamoxifen-induced endometrial pathology. Tsai et al. [50] showed that both estradiol and tamoxifen induce the cell migration of endometrial cancers with low or no nuclear ERα, through GPER1 activation. 

Other mechanisms possibly involved in tamoxifen’s action include the unfolded protein response (UPR) pathway, mTOR signaling, calcyphosin, and stathmin [6,51]. 

## 10. How to Explain the Janus-Headed Actions of Tamoxifen

In ERα-positive metastatic breast and endometrial cancer, tamoxifen treatment initially blocks the mitogenic actions of estrogens and leads to tumor regression. Some breast cancers and a large proportion of ECs have primary resistance to tamoxifen. Those that initially respond eventually become resistant to tamoxifen via several mechanisms, including the expression of GPER1, which mediates a stimulatory action of tamoxifen and fulvestrant. The latter finding might explain why fulvestrant is not superior to tamoxifen in the treatment of advanced EC [7,39,40]. 

When women take tamoxifen as an adjuvant treatment or for the prevention of breast cancer, some experience a relapse or new invasive breast cancer, indicating that some cells have become resistant to tamoxifen. In the endometria of women on adjuvant or preventive tamoxifen, long-term exposure (>2 years) is likely to offer a growth advantage to endometrial cells with pre-existing mutations [6]. This explains why postmenopausal women and those with the typical risk factors for EC including obesity are at a higher risk of developing tamoxifen-associated EC than premenopausal women, especially when they have no risk factors. It also explains the high incidence of endometrial pathology found before the initiation of tamoxifen treatment in postmenopausal breast cancer patients [28]. Tamoxifen might induce its growth-promoting effects in endometrial pathologies through a partial agonism of ERα [6] or through GPER1, which is overexpressed after chronic tamoxifen treatment (Table 1) [6,46,47,48,49,50].

## 11. Conclusions

Tamoxifen can induce EC during chronic exposure (adjuvant or preventive therapy) at a low frequency, preferably in postmenopausal women and those with typical risk factors for EC. This risk can be minimized by detecting and treating endometrial pathologies before the initiation of tamoxifen treatment [28]. The reduction of breast cancer mortality by adjuvant tamoxifen therapy far outweighs the slightly increased risks of EC. 

For patients with advanced or recurrent EC, tamoxifen alone or in combination with a progestogen is an efficacious treatment option with low toxicity [7,34,40]. 

The molecular mechanisms for the Janus-headed activity of tamoxifen (estrogen-antagonist/estrogen-agonist) are still elusive. Tamoxifen can act as an agonist or antagonist through ERα depending on cellular differences in co-activators or co-repressors. Tamoxifen acts as an estrogen agonist through GPER-1, which is more highly expressed in breast and endometrial cancer cells that show primary or secondary resistance to tamoxifen. As tamoxifen is and will be one of the most valuable anticancer drugs due to its high efficacy, low toxicity, and availability, further research to elucidate its mode of action should be encouraged. 

## Figures and Tables

**Table 1:** Proposed molecular mechanisms (selection) of tamoxifen in endometrium and endometrial (pre-) cancer [6].

**Mechanisms Stimulating Endometrial Proliferation and Development of EC**
- proliferative effects of tamoxifen through ERα - proliferative effects of tamoxifen mediated through GPER1 - unfolded protein response pathway, mTOR-signaling, calcyphosin, stathmin [6,51] - tamoxifen induced DNA damage?
**Mechanisms Mediating Therapeutic Effects of Tamoxifen in Advanced/Recurrent EC**
- estrogen antagonistic effects of tamoxifen on ERα - stimulation of expression of progesterone receptors through estrogenic effects of tamoxifen on ERα
